# Detection of rice sheath blight using an unmanned aerial system with high-resolution color and multispectral imaging

**DOI:** 10.1371/journal.pone.0187470

**Published:** 2018-05-10

**Authors:** Dongyan Zhang, Xingen Zhou, Jian Zhang, Yubin Lan, Chao Xu, Dong Liang

**Affiliations:** 1 Anhui Engineering Laboratory of Agro-Ecological Big Data, Anhui University, Hefei, Anhui, China; 2 Texas A&M AgriLife Research Center, Texas A&M University System, Beaumont, Texas, United States of America; 3 College of Resources and Environment, Huazhong Agricultural University, Wuhan, Hubei, China; 4 College of Engineering, South China Agricultural University, Guangzhou, Guangdong, China; Fujian Agriculture and Forestry University, CHINA

## Abstract

Detection and monitoring are the first essential step for effective management of sheath blight (ShB), a major disease in rice worldwide. Unmanned aerial systems have a high potential of being utilized to improve this detection process since they can reduce the time needed for scouting for the disease at a field scale, and are affordable and user-friendly in operation. In this study, a commercialized quadrotor unmanned aerial vehicle (UAV), equipped with digital and multispectral cameras, was used to capture imagery data of research plots with 67 rice cultivars and elite lines. Collected imagery data were then processed and analyzed to characterize the development of ShB and quantify different levels of the disease in the field. Through color features extraction and color space transformation of images, it was found that the color transformation could qualitatively detect the infected areas of ShB in the field plots. However, it was less effective to detect different levels of the disease. Five vegetation indices were then calculated from the multispectral images, and ground truths of disease severity and GreenSeeker measured NDVI (Normalized Difference Vegetation Index) were collected. The results of relationship analyses indicate that there was a strong correlation between ground-measured NDVIs and image-extracted NDVIs with the *R*^*2*^ of 0.907 and the root mean square error (RMSE) of 0.0854, and a good correlation between image-extracted NDVIs and disease severity with the *R*^*2*^ of 0.627 and the RMSE of 0.0852. Use of image-based NDVIs extracted from multispectral images could quantify different levels of ShB in the field plots with an accuracy of 63%. These results demonstrate that a customer-grade UAV integrated with digital and multispectral cameras can be an effective tool to detect the ShB disease at a field scale.

## Introduction

Rice is one of the most important food crops in the world, providing a major source of nourishment for over half the world population. Rice diseases, especially sheath blight (ShB) caused by *Rhizoctonia solani* AG1-1A, are among the most important factors limiting rice production worldwide [1]. The ShB disease usually develops in the later tillering or early internode elongation stage of rice. Infected tissue looks brown to yellow or even bleached in color. Symptoms of the disease first appear on the lower sheaths of plants and then develop onto upper sheaths and leaves. ShB spreads from plant to plant through the growth of the fungus and usually forms in a circular pattern in the field [2]. Under favorable conditions, this disease spreads quickly to top plant parts, causing lodgings of the plants. The disease occurs every year, causing significant losses in grain yield and quality in the US and other rice-producing counties of the world [2]. ShB has become the second most economically-important disease in rice in the world [2].

Cultivar resistance can be the most effective, economic means for management of rice ShB and other crop diseases. Plant phenotyping plays a critical role in breeding programs for the development of cultivars of rice and other field crops with improved disease resistance. Plant phenotyping also has been widely used in crop breeding programs to assess plant traits such as plant growth, development, abiotic stress tolerance, architecture, physiology, ecology, and yield [4,5]. Recently, optical sensor technologies have been developed and used to assist plant phenotyping and disease detection and diagnosis [6]. Digital (red, green, and blue)-, multispectral-, hyperspectral-, flrorescence imaging- and thermal infrared-based sensors have been utilized to characterize plant disease symptoms, detect different diseases, and even quantify severity of several diseases in the laboratory and field [7,8]. These studies have been conducted on wheat, barley and vegetables including tomatoes and cucumbers [6]. However, no significant advances in high-throughput phenotyping have been made on rice. This is probably due to the flooding conditions under which rice plants grow, limiting the ability to use this remote sensing technology.

Unmanned Aerial Systems, one of new remote sensing platforms, has played an important role in application in precision agriculture. This technology has the advantages of high-spatial resolution, high efficiency, low costs, and flexibility in use [9–11]. Use of the technology can facilitate the quick and accurate detection of plant diseases at a field scale and thus improve disease management efficacy through on-time applications and/or site-specific applications of fungicides.

In this study, a quadrotor unmanned aerial vehicle (UAV), equipped with digital (Red, Green, and Blue bands) or multispectral (Near-infrared, Red edge, Red, Green to Blue bands) camera, was utilized to capture high-spatial resolution imagery data of different ShB-resistant rice cultivars and lines in research plots to characterize and detect the ShB disease in rice at a field scale. The objectives of this work were to: 1) quantify the changes in color, texture and structure associated with the symptoms of ShB at the level of canopy by high-resolution digital and multispectral images, 2) determine the correlations between color features or vegetation indices and ShB severity, and 3) evaluate the advantages and disadvantages of vegetation indices-based detection of ShB.

## Materials and methods

### Experimental site

A field trial was conducted at Texas A&M AgriLife Research Center (30°03'53.4"N, 94°17'38.7"W), Beaumont, Texas, USA. Sixty-seven rice cultivars and elite lines with different levels of resistance to ShB (Table 1) were selected to characterize and detect their differences in the development of the disease in research plots. Rice was drill seeded in 67 plots consisting of seven 2.4-m rows, spaced 18 cm between rows on May 8, 2015. Each plot was divided into two equal-length sections. One end section of the plot was inoculated with the ShB pathogen by manually broadcasting 100 ml of the *R*. *solani* inoculum on July 10; the other end section was left with no pathogen inoculation, serving as the disease-free control. Our previous observations showed that the ShB pathogen has very limited ability to spread from one section to another [12]. This experiment was specifically designed to allow for observing and comparing the development of the disease at different growth stages of rice. Fertility, irrigation, and weed and insect pest control followed local recommendations [2]. On August 23 and 30, the severity of ShB was rated on a scale of 0 to 9 where 0 represents no symptoms and 9 represents most severe in symptoms and damage (leaves dead or plants collapsed). The resistance of selected cultivars to ShB was indicated as very susceptible (VS), susceptible (S), and moderately susceptible (MS) (Table 1). Both disease assessment dates corresponded approximately 3 and 2 weeks from maturity of most of the rice cultivars and elite lines evaluated in this study.

**Table 1:** Severity of sheath blight (ShB) in 67 rice cultivars and lines in the field trial at Beaumont, Texas, USA.

Cultivar/line	Resistance*	ShB severity (0–9)		Cultivar/line	ShB severity (0–9)	
		Aug 23^rd^	Aug 30^th^		Aug 23^rd^	Aug 30^th^
Antonio	VS	4.0	5.3	RU120-2	8.0	8.3
Cheniere	VS	5.0	7.0	RU130-1	4.3	5.5
CL111	VS	7.7	8.4	RU130-2	4.0	6.0
CL151	S	7.8	8.1	RU130-3	6.0	6.3
CL152	S	8.0	8.3	RU130-4	4.0	5.3
CL163		6.7	7.0	RU130-5	6.0	8.0
CL271	MS	4.7	5.3	RU130-6	4.0	6.3
CLXL729	-	4.0	5.2	RU130-7	5.0	8.0
CLXL745	-	4.0	5.2	RU130-7	7.0	7.7
Cocodrie	VS	7.9	8.1	RU130-8	5.0	7.3
Colorado	VS	8.0	8.3	RU140-1	7.0	7.7
Della-2	MS	4.0	5.3	RU140-10	6.8	7.0
Jazzman-2	S	8.2	8.4	RU140-2	6.7	7.0
Jupiter	S	5.9	6.1	RU140-3	4.0	5.0
LAH10	-	4.0	5.3	RU140-4	4.0	5.0
LAH169	-	4.8	5.5	RU140-5	7.0	7.0
Mermentau	-	6.8	7.1	RU140-6	4.0	5.3
Presidio	VS	7.0	7.3	RU140-7	5.0	6.7
Rex	S	3.0	5.0	RU140-8	5.3	7.0
Rondo	MS	3.0	4.7	RU140-9	8.0	8.3
RU070-1	-	3.0	5.0	RU150-1	6.0	7.0
RU080-1	-	4.0	7.0	RU150-10	6.0	8.0
RU080-2	-	9.0	9.0	RU150-2	7.0	8.0
RU080-3	-	8.0	8.3	RU150-3	5.7	6.0
RU080-4	-	8.0	9.0	RU150-4	5.0	7.3
RU090-1	-	3.0	5.3	RU150-5	8.0	9.0
RU090-2	-	6.7	7.0	RU150-5	5.7	6.0
RU090-3	-	5.0	7.3	RU150-6	8.3	9.0
RU090-4	-	5.0	5.3	RU150-7	4.0	5.0
RU100-1	-	7.7	8.0	RU150-8	3.0	5.3
RU100-2	-	7.0	8.3	RU150-9	6.7	8.0
RU100-3	-	6.0	6.7	XL753	3.3	5.0
RU100-4	-	5.3	7.8	XP760	2.8	5.0
RU120-1	-	7.0	8.0			

* VS = Very susceptible, S = Susceptible, and MS = Moderately susceptible.

Original field data in Table 1 are included in the files S1 Table, S1 Fig and S2 Fig in the Support Information.

### Unmanned aerial system

The 4-rotor UAV equipped with high-resolution digital or multispectral camera was used to collect imagery data in the research plots. The UAV is the Phantom 2 Vision+ (Da-Jiang Innovations Science and Technology Co., Ltd, Shenzhen, China) with the advantages of stable 3-axis gimbal, automatically GPS (Global Position System) data recording, and user-friendly flight control. The digital camera offers the image quality of 14 Megapixels with the size of the 4384×3288 pixel array for Blue, Green and Red bands. The multispectral camera Micasense RedEdgeTM (MicaSense, Inc., Seattle, WA, USA) can acquire 12-bit raw image in five narrow bands from blue, green, red, red edge to near-infrared (NIR). The 5-channel camera could measure plant reflectance to capture subtle information about crop stress more accurately than the regular 3-channel (blue, green, and red) camera [13].

### Data collection and processing

#### Image capture

The digital and multispectral cameras were used to capture high-spatial resolution images over the field plots. Before imagery data were collected, optimal exposure time for different cameras was selected based on weather conditions; the actual parameters were set according to the instructions of the software [13,14]. During the flights, the cameras were positioned at the nadir at two altitudes, 27 m above the ground to cover all 67 experimental plots and 5.5 m to cover four plots in each image. The appropriate flight overlaps were adjusted by different flying heights. In order to stably capture the images, the UAV was instructed to fly along the experimental plots with the wind direction and the flights were set at a speed between 0 and 10 m/s depended on wind speed. The flight experiments were conducted from 12:30 pm to 2:00 pm on Aug 23^rd^ and 30^th^. The weather conditions were favorable for the flights, with partly cloudy and breeze condition during the flights.

These imagery data are included in the S1 File in the Support Information File.

#### Image processing

The ENVI (Exelis Visual Information Solutions, Boulder, CO, USA) software was used to extract color features from the acquired images and convert them to different color spaces. Previous studies demonstrate that color space transformation can improve the presentation of information, and the transformed images can be interpreted more easily than the original ones [15]. In this study, the digital images (Red, Green and Blue bands) of different levels of ShB severity were transformed to hue, lightness and saturation (HLS), and then the mean values of HLS were extracted.

#### Vegetation indices calculation

Five kinds of vegetation indices (VIs), including Normalized Difference Vegetation Index (NDVI), Ration Vegetation Index (RVI), Difference Vegetation Index (DVI), Normalized Difference Water Index (NDWI) and Red Edge (RE), were calculated from the acquired multispectral images. And then, the NDVIs-change maps of different levels of disease severity were generated so as to illustrate that multispectral imagery data could detect the symptoms and development of the ShB disease at a field scale.

#### Collection of ground truths

Ground-based NDVI values of rice cultivars and lines were measured by GreenSeeker handheld crop sensor (Trimble Navigation Limited, California, USA). The operating mechanism of the sensor is based on the fact that green plants absorb most of the red light and reflect most of the infrared light. The relative strength of the detected light is a direct indicator of the density of the foliage within the sensor view. The denser and more vigorous the plant, the greater the difference is observed between the reflected light signals. When taking the ground NDVI readings in this study, the GreenSeeker handheld crop sensor was held 100 cm above the canopy of rice plants, with an ovalfield of view covering the area of 42 cm^2^. Multiple measurements were taken in each plot to increase the accuracy of the NDVI values that were representative of the levels of ShB severity. A total of 134 NDVI average readings (the relative ground truths) were collected from the pathogen-inoculated and un-inoculated control areas of 67 plots in the trial. On the same assessment dates, severity of ShB was rated at a scale of 0–9 based on the symptoms of the disease as described before.

#### Data analyses and drawing

The Pix4D mapper (Pix4D Inc, Lausanne, Switzerland) was used for UAV image processing. ArcGIS 9.1 (Esri, Redlands, CA, USA) was used for geospatial data analysis and mapping. PASW Statistic 18 (SPSS Inc., Chicago, IL, USA) software was used for statistical analyses. Determination coefficient and root mean square error (RMSE) were utilized to evaluate the accuracy of correlation model [16].

## Results and analyses

### Qualitative ShB detection based on high-resolution images

#### Characterizing ShB at two development stages

In this study, color was selected as the most important characteristic to detect the ShB disease. This is because infected plant tissue usually changes its color from green (healthy tissue) to brown-to-yellow (diseased tissue) with the development of the disease. Color has been demonstrated as the most effective means to distinguish different image targets and achieve object identification among the morphological features, such as color, texture, size, etc. extracted from images in previous studies [17,18].

**Fig 1:**
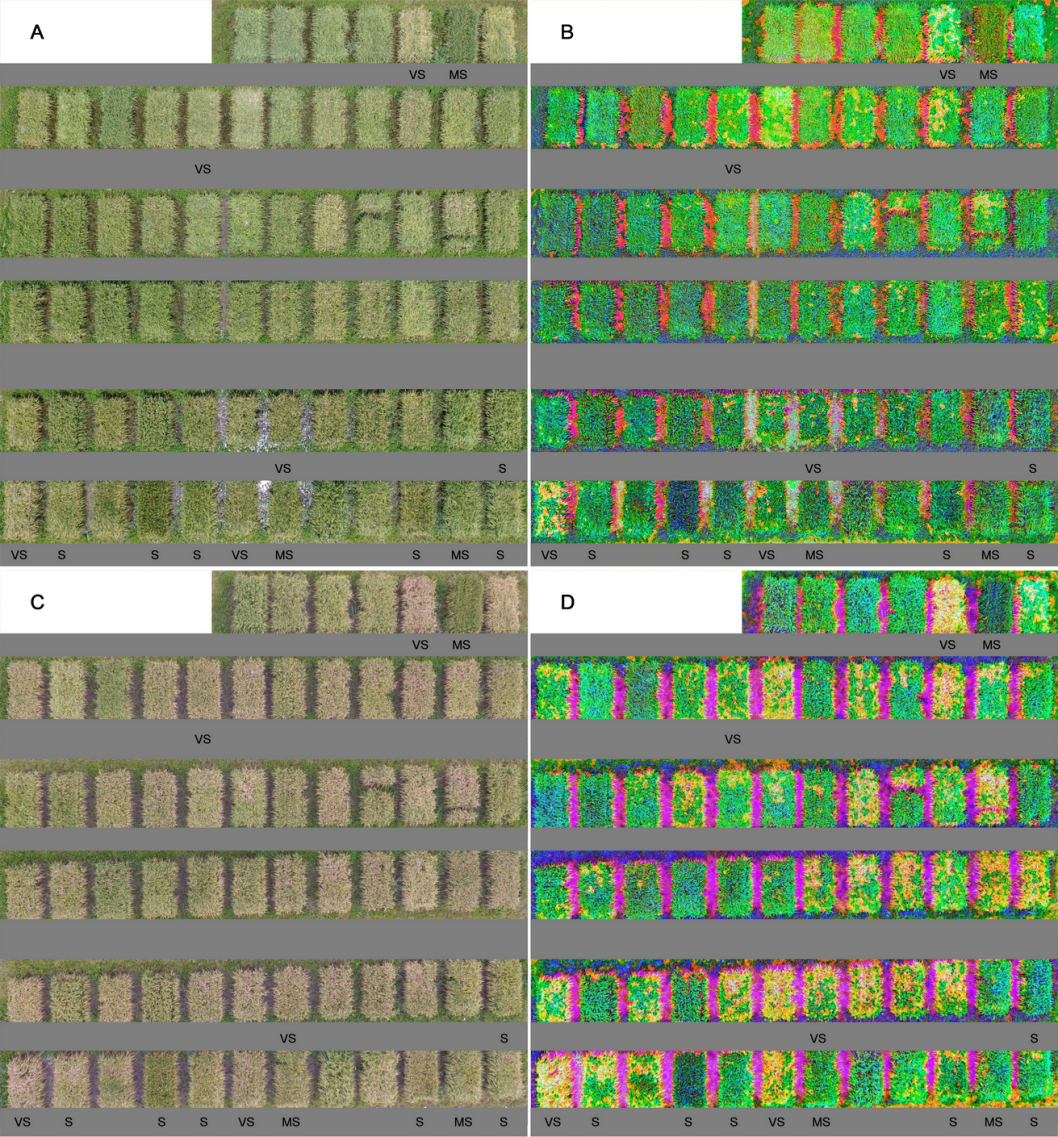
**Original RGB and HLS transformation images of 67 field plots on Aug 23**^**rd**^ **(A and B) and Aug 30**^**th**^**(C and D).** The ratings of resistance to ShB with very susceptible (VS), susceptible (S), and moderately susceptible (MS) were indicated in selected plots (cultivars or lines).

As shown in Fig 1A and 1C, the color of rice canopy in the RBG images of field plots changed significantly with the growth of rice plants. However, no significant differences in color change were observed between ShB-infected and uninfected control areas in most of 67 plots at either assessment date. Previous studies demonstrate that the illumination factor produces color features difference of the images, but color space transformation can eliminate illumination difference and strengthen color features such as hue, lightness, and saturation for different targets [15]. Therefore, RGB images were transformed to HLS images to differentiate the ShB-infected areas. The images in Fig 1B and 1D clearly showed yellow ShB-infected areas and green or blue healthy areas for all plots at either observation date. Moreover, these differences in images were more apparent at the Aug 30^th^ assessment date (Fig 1D) than at the Aug 23^rd^ assessment date (Fig 1B) although they were only seven days in apart. Meanwhile, these differences were more obvious in plots with susceptible cultivars and lines than in plots with resistant or partially resistant ones. These results illustrate that HLS-transformed color space is a useful means to qualitatively detect rice ShB in the field.

#### Comparison of ShB detection using RGB and multispectral images

**Fig 2:**
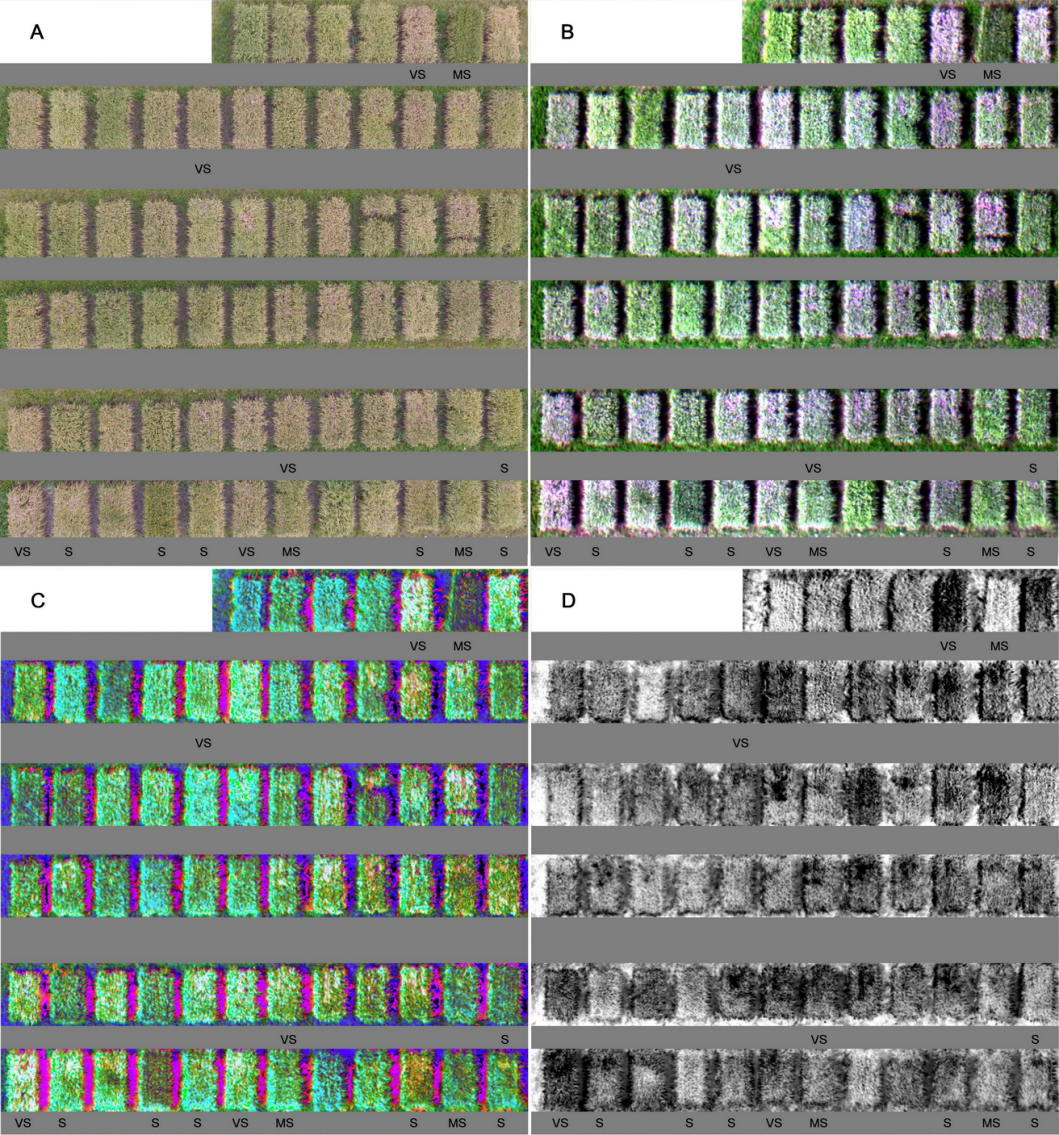
**RGB, False, HLS, and NDVI images of 67 field plots on Aug 30**^**th**^ **(A, B, C and D).** The ratings of resistance to ShB with very susceptible (VS), susceptible (S), and moderately susceptible (MS) were indicated in selected plots (cultivars or lines).

It is well known that multispectral cameras can provide good service to precision agriculture management such as disease and pest detection, drought monitoring, nutrition diagnosis, and spray drift evaluation [19–22]. In this study, high-resolution RGB and 5-bands multispectral images were analyzed to detect ShB-infected areas in the field plots. As shown in Fig 2A and 2B, the imagery data collected from multispectral camera could reflect field environments (green weed plants, ground earth, plots shadow, etc.) and canopy characteristics (color, texture, and structure information, etc.) more accurately. When the false color image (Fig 2B) was transformed into HLS combination (Fig 2C), it resulted in more apparent display of the ShB-affected areas with yellow to white in color in the 67 experimental plots. In addition, the NDVIs map of rice cultivars and lines in the field plots was also developed (Fig 2D) after NDVI values were calculated. The darker the image color, the more severe the ShB disease. In Fig 2D, the diseased areas were clearly differentiated from the healthy areas in each of the plots. These differentiations were more effective compared to the differences made by RGB, False, or HLS images (Fig 2A, 2B and 2C). It can be explained that red and near-infrared lights are more sensitive to the changes in canopy color from green (healthy) to yellow (diseased) and the changes in canopy structure from dense to sparse in density caused by the development of ShB [3,19]. Therefore, the vegetation index NDVI is a good indicator of different levels of ShB observed in this study.

### Quantitative ShB detection based on color feature parameters

#### Correlations between color features and ShB severity

The regular RGB has been used to quantitatively detect disease and insect pests, and other crop stresses caused by drought and nitrogen deficiency in agricultural production systems in previous studies [23–25]. In this study, quantitative detection of ShB was also evaluated based on high-resolution RGB images. The relationships between color features and disease severity were analyzed for both disease assessment dates (Table 2). The color features were extracted from red, green, blue bands, and color space transformed hue value. It is noted that the relationships between other color space transformed values (light and saturation) and ShB severity were insignificant, so we only presented the results of hue values in Table 2.

**Table 2:** Relationships between color features and ShB severity.

Color features	Assessment date	Calculation equation	R^2^	RMSE
Red band	Aug 23^rd^	y = 0.017x+3.3356	0.038	19.638
Green band	Aug 23^rd^	y = 0.0185x+2.9465	0.056	15.957
Blue band	Aug 23^rd^	y = 0.0223x+3.4965	0.073	19.331
Hue	Aug 23^rd^	y = -0.004x+5.7393	0.010	40.831
Red band	Aug 30^th^	y = 0.0522x+1.3841	0.251	12.633
Green band	Aug 30^th^	y = 0.0168x+4.3868	0.011	7.961
Blue band	Aug 30^th^	y = 0.0342x+2.9826	0.196	17.044
Hue	Aug 30^th^	y = 0.0143x+4.6117	0.554	68.376

As shown in Table 2, the determination coefficients (R^2^) were significantly low with a value ranging from 0.038 to 0.251 for color features of RGB image at both disease assessment dates. However, R^2^ value for color space hue on Aug 30^th^ was 0.554, which was obviously higher than other color features. Therefore, RGB-based color features are less effective to quantitatively detect different levels of ShB, but color space transformation can improve its ability to quantify the severity of the disease.

#### Correlations between VIs and ShB severity

Vegetation index is considered as a simple and effective quantitative parameter to monitor the growth status and coverage of green vegetation on the earth surface [26]. It has been extensively used to determine disease severity, nutrition status, drought stress, and yield of several crops [27–29]. In this study, five kinds of vegetation indices, NDVI, RVI, DVI, NDWI and RE, were chosen to determine their ability to quantify ShB severity. NDVI had the highest R^2^ value (0.627) with the lower RMSE (0.0854) compared to those of other VIs (Table 3). NDVI was also superior to all the color features evaluated in this study (Table 2). Thus, these results indicate that NDVI has the best performance on the detection of different levels of ShB in the field plots. This is because NDVI is sensitive to the changes in the density of ground vegetation from high to low due to the damage caused by the disease, and the changes in canopy color from green to yellow or white caused by plant growth toward maturity [26]. For the other VIs, RVI is able to monitor the growth status of green vegetation but its sensitivity decreases significantly when canopy coverage reduces to less than 50% [28]. DVI is a good vegetation index to detect low-to-moderate levels of vegetation coverage but it is more likely to be affected by backgrounds such as soil, water and shadow [29]. NDWI is sensitive to the changes in liquid water content of vegetation canopies. However, it can be affected by residual irrigation water in field plots [30]. RE is able to detect accurately the changes in leaf chlorophyll content and biochemical components of plant. Its sensitivity, however, decreases significantly when the crop approaches the late stages of growth.

**Table 3:** Correlations between VIs and ShB severity.

VIs	Assessment date	Calculation equation	R^2^	RMSE
NDVI	Aug 30^th^	y = -0.0513x+0.479	0.627	0.0852
RVI	Aug 30^th^	y = -2.0782x+9.6484	0.1336	0.231
DVI	Aug 30^th^	y = -7E-05x+7.4872	0.0954	55.059
NDWI	Aug 30^th^	y = 17.929x+7.5417	0.0658	0.018
RE	Aug 30^th^	y = 2.5935x+4.8018	0.1413	0.191

## Discussion

### Influence of spectral bands and their combination on the detection of ShB

Previous studies indicate that digital image can be a good data resource to detect crop diseases [27–29]. In this study, high-resolution RGB and multispectral imagery data were acquired and analyzed with the aim of qualitatively and quantitatively detecting the rice ShB in research plots. The results demonstrate that color features extracted from RGB and multispectral images could partially distinguish the canopy changes caused by the disease. However, it does not provide sufficient information to differentiate different levels of ShB because of less spectral wavelength and broad bands associated with the RGB camera used in this study. This limits the potential application of UAV equipped with RGB camera to the detection of the disease at a commercial scale and to the screening of ShB-resistant cultivars and elite lines in research plots for the rice breeding program.

**Fig 3:**
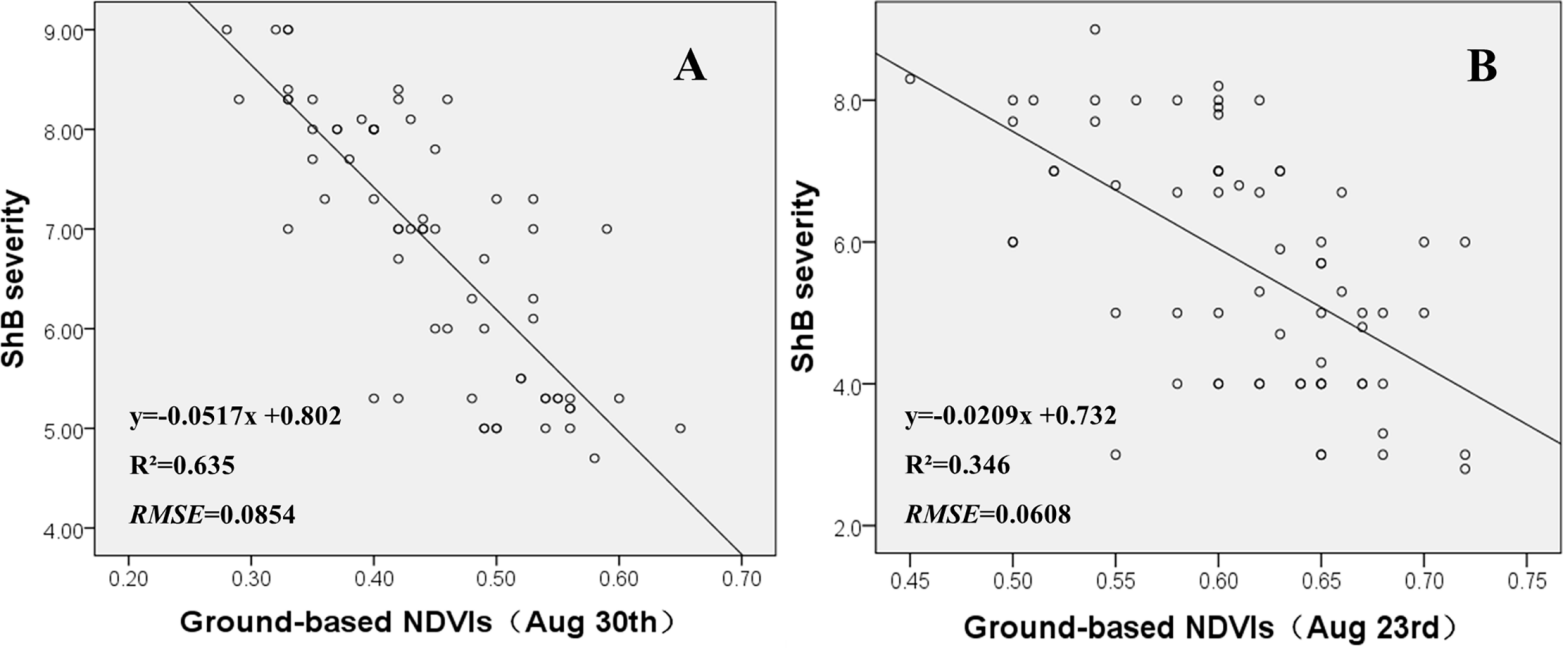
**Correlations between ground-based NDVIs and ShB severity on Aug 30^th^ (A) and Aug 23^rd^ (B)**.

**Fig 4:**
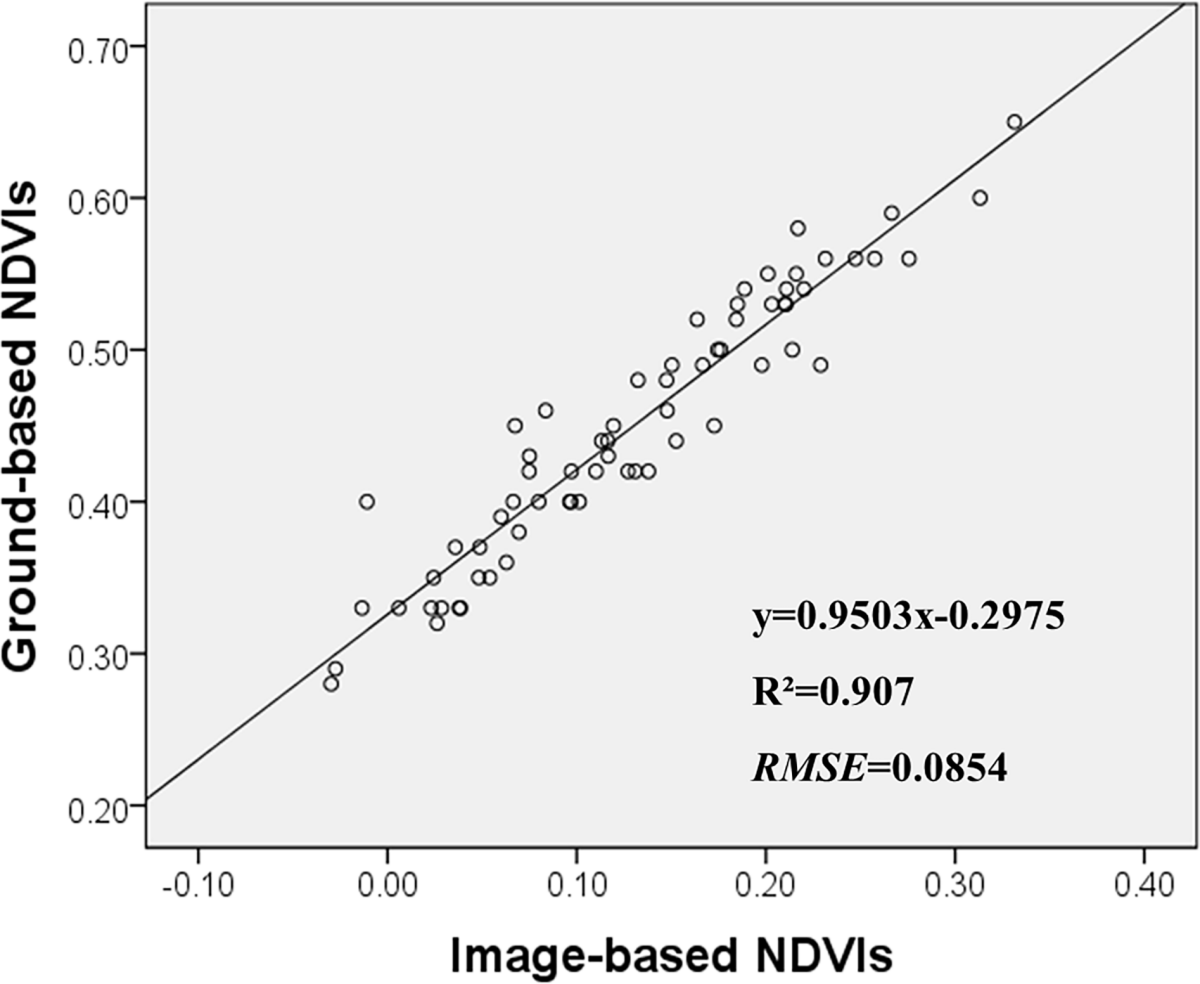
Correlation between image-based NDVIs and ground-based NDVIs on Aug 30^th^.

On the contrary, NDVI, one of five kinds of VIs calculated from multispectral imagery data in this study, had a strong relationship with ShB severity. The R^2^ and RMSE values are, respectively, 0.627 and 0.0852 for the correlation between image-based NDVIs and ShB severity (Table 3), and 0.635 and 0.0854 for the correlation between ground-measured NDVIs and ShB severity (Fig 3A). Meanwhile, we also analyzed the correlation between ground-measured NDVIs and image-based NDVIs, the R^2^ and RMSE values are, respectively, 0.907 and 0.0854 (Fig 4). These results demonstrated that NDVIs calculated from multispectral imagery data could provide better spectral information to differentiate different levels of ShB in the field plots.

In addition, the correlations between ground-measured NDVIs and ShB severity for different growth stages were compared. The R^2^ and RMSE values are, respectively, 0.635 and 0.0854 on Aug 30^th^ (Fig 3A), and 0.346 and 0.0608 on Aug 23^rd^ (Fig 3B). These results indicate that it is insufficient to utilize NDVIs to quantify ShB severity at an earlier stage of disease development. This is in agreement with the results obtained from previous studies [3,16]. More works is needed to improve the differentiation accuracy of rice ShB at early growth stages. Using hyperspectral sensor might be a way to characterize subtle details of crop diseases. Yang demonstrated that VIs calculated from nano-level hyperspectral bands can assess the severity of bacterial leaf blight in rice precisely [16]. Mahlein pointed out that hyperspectral camera and VIs derived from sensitive bands have data benefits to diagnose plant diseases and phenotypes [6]. Our previous research also confirmed that hyperspectral sensor with broad wavelengths and narrow bands can capture subtle changes in the symptoms of ShB in rice at an earlier stage of disease development [3]. Therefore, additional investigations are needed to explore the potential use of narrow-bands multispectral and hyperspectral sensors to effectively detect ShB at early stages of development.

#### Influence of sampling area selection on the detection of ShB

**Fig 5:**
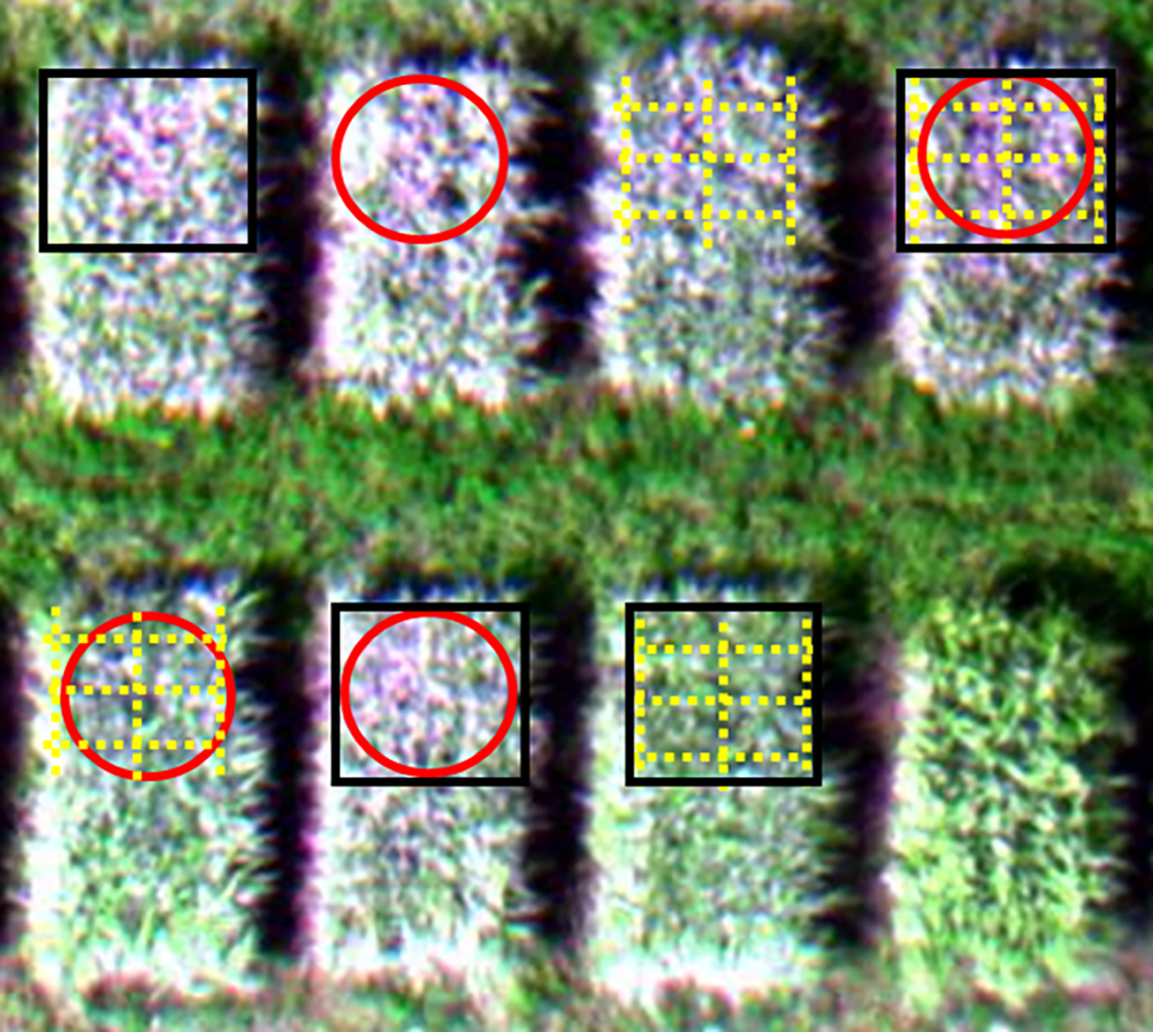
Three sampling methods. They were assigned as rectangle area sampling (black rectangle), circular area sampling (red circle), and manual sampling by a plant pathologist (yellow dashed lines).

**Fig 6:**
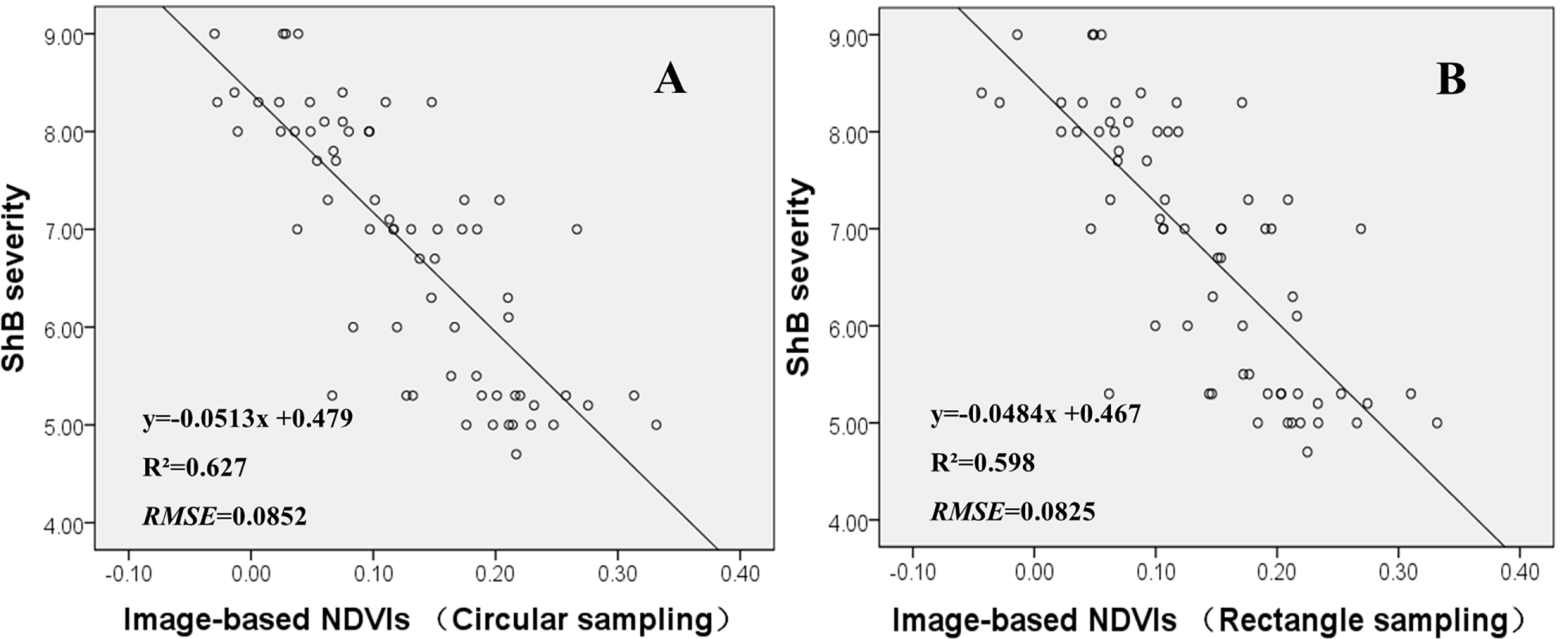
**Correlations between image-based NDVIs and ShB severity using circular sampling (A) and rectangle sampling (B) methods on Aug 30^th^**.

Selection of sampling areas in the field can affect the accuracy of detecting ShB severity due to the nature of cluster distribution of the disease in infected fields [2,3]. In this study, three methods of selecting a sample area, circular sampling, rectangle sampling, and manual selection by a plant pathologist, were evaluated to determine their efficacy of detecting ShB severity (Fig 5). These three sampling methods were all acceptable in efficacy with no significant differences in R^2^ and RMSE. The R^2^ and RMSE values for the correlation between image-extracted NDVIs and ShB severity are, respectively, 0.627 and 0.0852 for the circular area sampling method (Fig 6A), 0.598 and 0.0825 for the rectangle area sampling method (Fig 6B), and 0.635 and 0.0854 for the selection of sampling area by an investigator (Fig 3A). The reason for the method of selecting sample area by an investigator having a highest value of R^2^ is because such manual selection can effectively target diseased areas for disease assessment. The R^2^ value is relatively greater for the circular area sampling method than for the rectangle area sampling method. This is due to fact that the circular area sampling method could cover more percentage of diseased areas in the field plots than the later one. The rice ShB tends to spread more likely in circular pattern than in rectangle pattern from a point focus of inoculation in the field [2]. This resulted in a decreased error in disease detection made by the circular area sampling method. Therefore, selecting an optimal sampling method based on the spread pattern of a disease is also a factor to improve disease detection efficacy when using image-based UAV platforms.

### Influence of flying altitude on the detection of ShB

**Fig 7:**
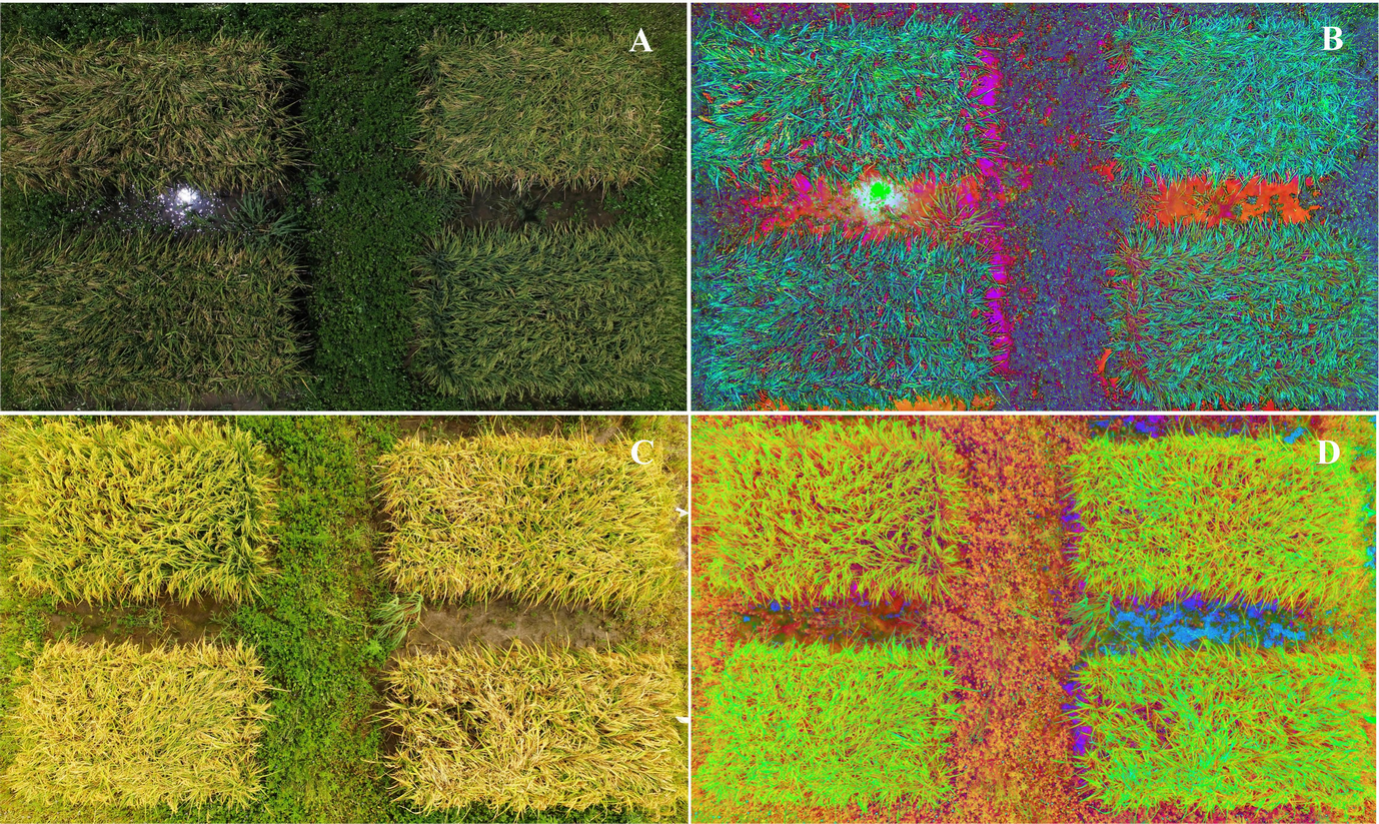
**RGB and HLS images at 5.5-m flying altitude on Aug 23^rd^ (A and C) and Aug 30^th^ (B and D)**.

Flying altitude can be an important factor affecting the ability of UAVs to detect the details of ShB development and plant canopy in the field. In this study, UAV imagery data at an altitude of 5.5 and 27 m above the ground were collected and evaluated on two assessment dates. At the altitude of 5.5 m, detailed information about the lodging caused by ShB, and canopy components such as leaf color, rice ears and other canopy structures were clearly displayed in original RGB (Fig 7A and 7C), but there was no significant difference in the transformed HLS images (Fig 7B and 7D) on Aug 23^rd^ and Aug 30^th^. At this altitude, however, quantitative analyses were not conducted for multispectral images since the flight height was too low to have the high quality of imagery data. At the altitude of 27 m (Figs 1 and 2), we were able to not only qualitatively analyze the color characteristics of rice canopy obtained from RGB- and multispectral cameras but also quantitatively evaluate the performance of NDVIs extracted from multispectral imagery on the detection of the rice ShB in the field. The results of this study indicate that such flying altitude was acceptable but further research is still needed to explore the optimum flying altitudes that can maximize the ability of UAV to detect ShB in rice.

## Conclusions

A commercial UAV equipped with a high-resolution RGB and multispectral cameras was used to capture imagery data. Collected imagery data were then processed and analyzed to characterize the development of ShB and quantify different levels of the disease in the field. Ground truth data of ShB severity and NDVIs were also measured for comparisons. Hue value of color features obtained from RGB images can clearly differentiate the infected areas from the healthy, uninfected areas in field plots on August 30^th^. NDVIs calculated from the multispectral images could quantify different levels of the disease in the plots with an accuracy of 63%. Image-based NDVI values were strongly correlated with ground-NDVI values with R^2^ of 0.91. There was a good relationship (R^2^ = 0.64) between ground-NDVI values and disease severity. These results demonstrate for the first time that image-based NDVI is an effective means to detect ShB and quantify the severity of the disease at a field scale.

Combined use of an UAV with high-spatial resolution camera is an innovation that has the high potential for quick and accurate detection of ShB, one of the most important diseases in rice in the world. This technology can aid in the scouting and monitoring process of this disease and reduce the costs in time and effort associated with this process. This UAV system in the current form can also assist crop breeders in breeding for rice cultivars with resistance to ShB. In addition, such new UAV system developed from this research also has provided a basis to develop site-specific precision fungicide application technology for control of this important disease in rice in the future.

## Supporting information

S1 Table Disease assessment data. (XLSX)

S1 Fig Ground truth data (0823). (JPG)

S2 Fig Ground truth data (0830). (JPG)

S1 File Imagery data (0823–0830). (DOCX)
