# Supplementary figures and images for: Detection of rice sheath blight using an unmanned aerial system with high-resolution color and multispectral imaging
Source: PLoS One. 2018 May 10;13(5):e0187470. doi: 10.1371/journal.pone.0187470 (PMC5945033; doi:10.1371/journal.pone.0187470)

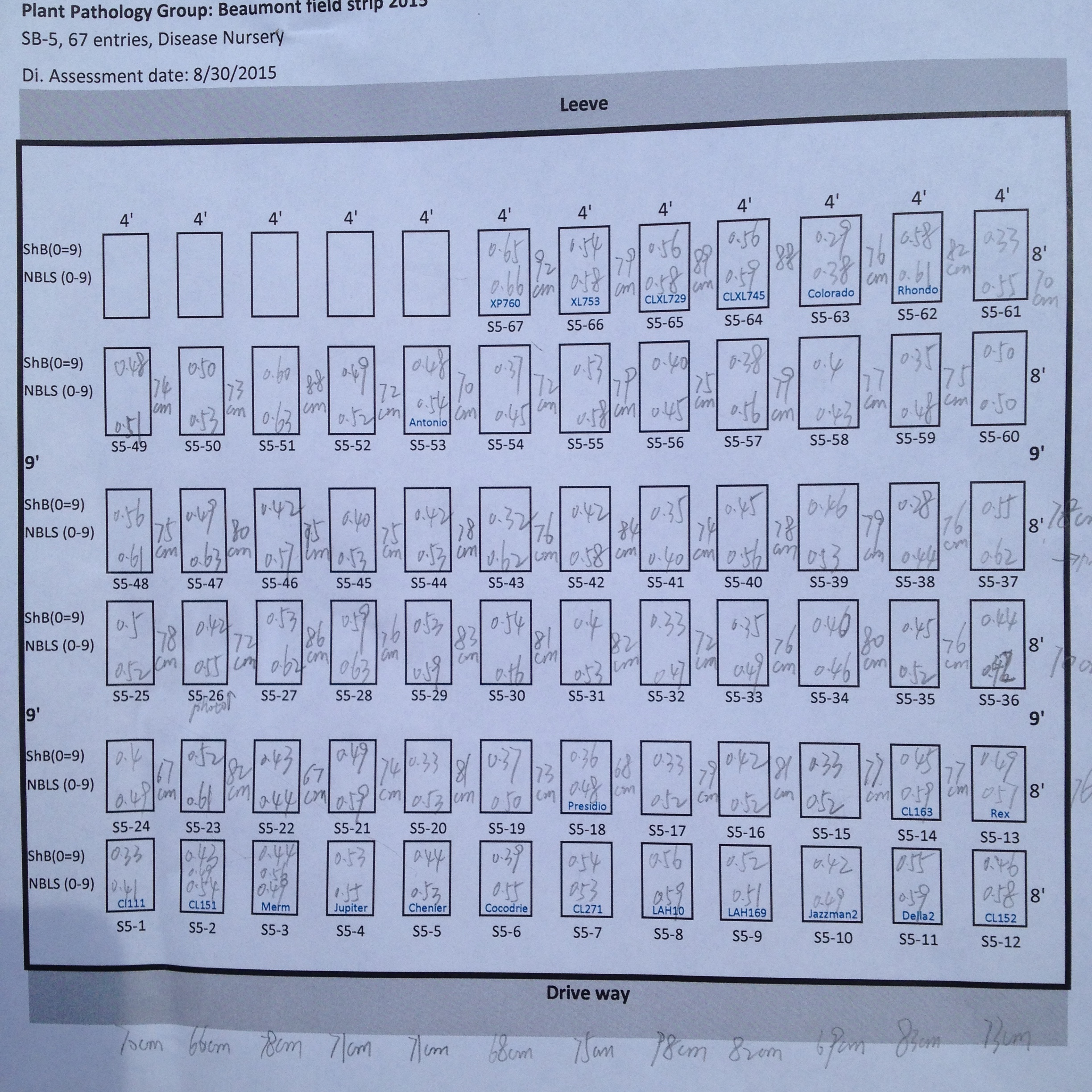

Supplement: S1 Fig — (JPG) [file pone.0187470.s002.JPG]

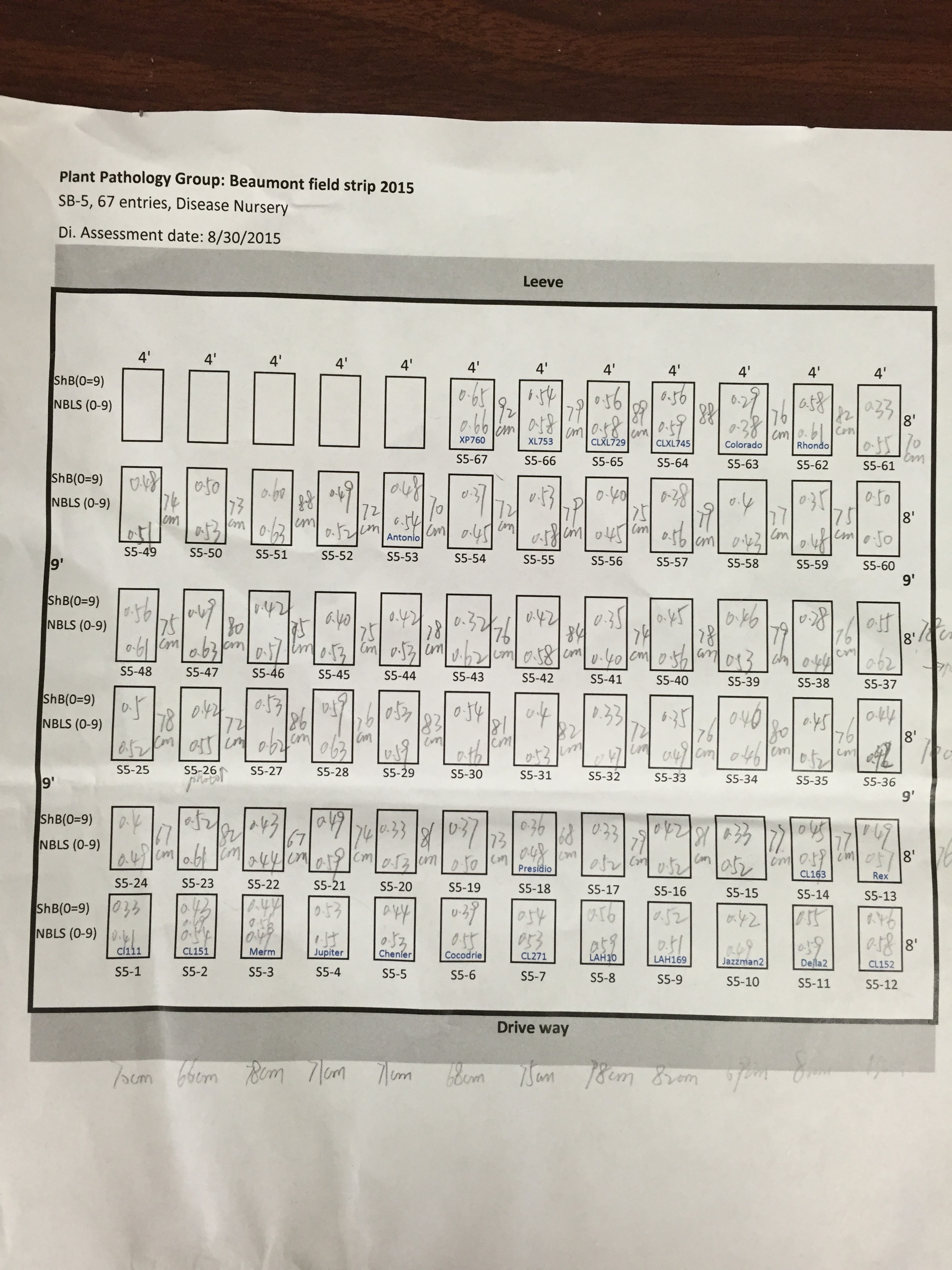

Supplement: S2 Fig — (JPG) [file pone.0187470.s003.JPG]
