# Supplementary material for: Detection of rice sheath blight using an unmanned aerial system with high-resolution color and multispectral imaging
Source: PLoS One. 2018 May 10;13(5):e0187470. doi: 10.1371/journal.pone.0187470 (PMC5945033; doi:10.1371/journal.pone.0187470)
Supplement: S1 File — (DOCX) [file pone.0187470.s004.docx]

**
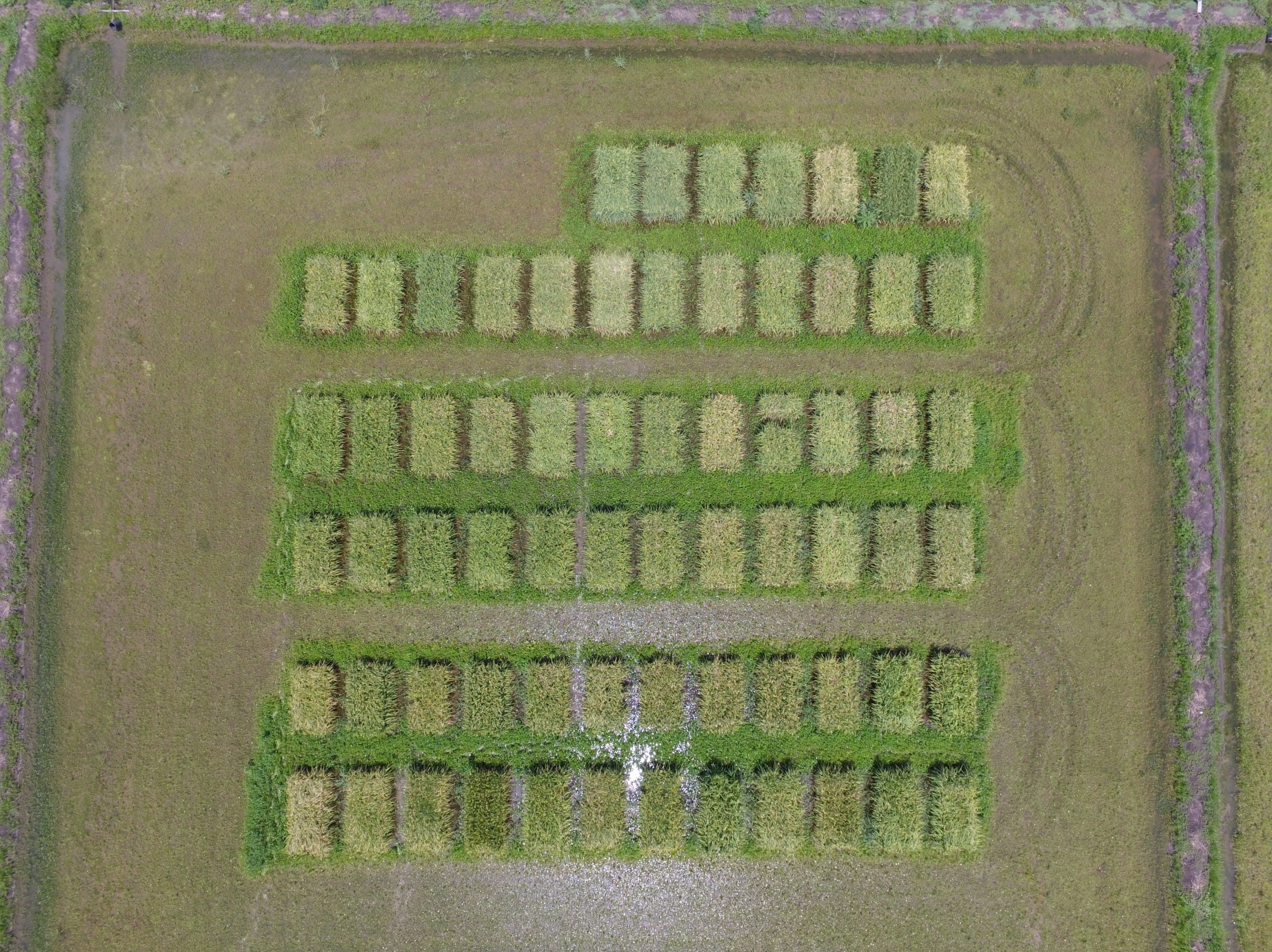
**
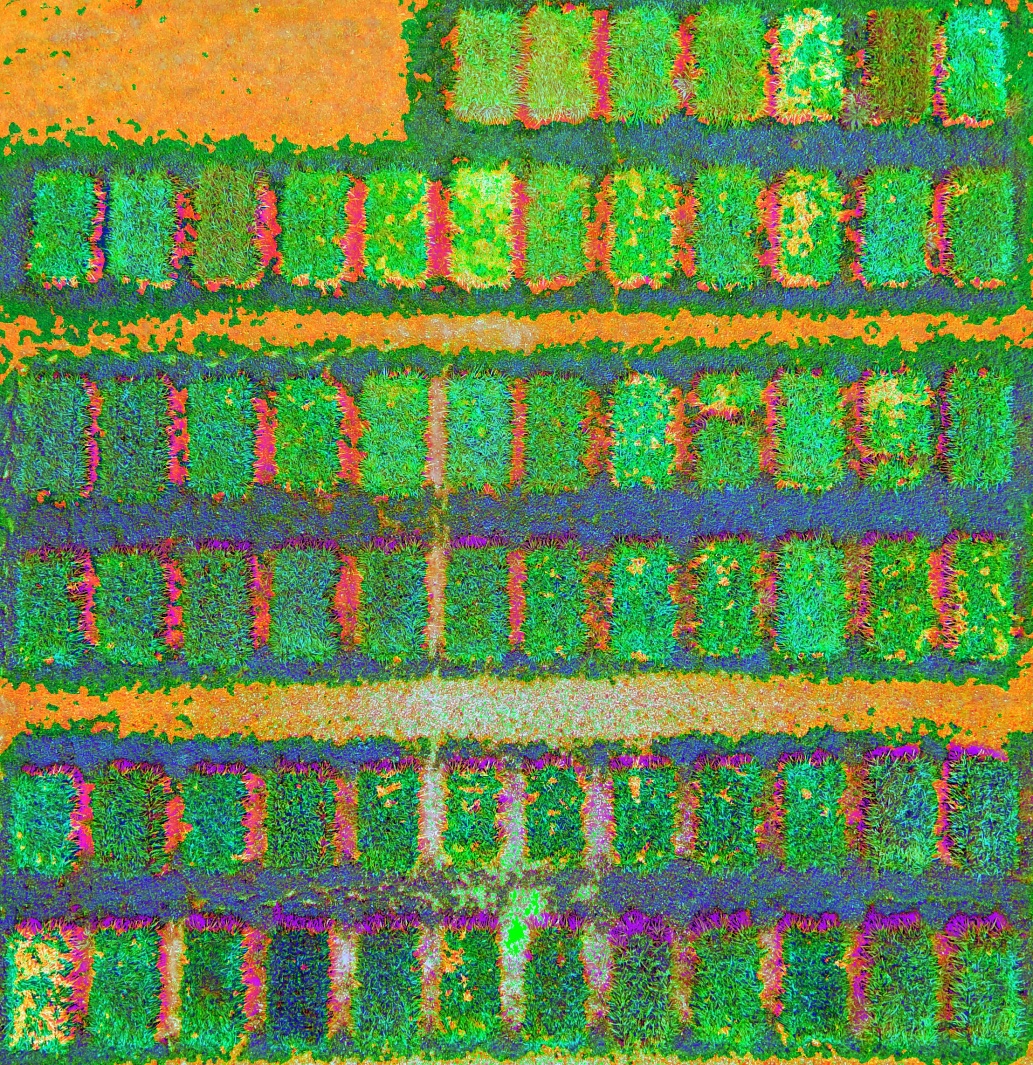


**（a） RGB image （b） HLS image**

**First data collection**


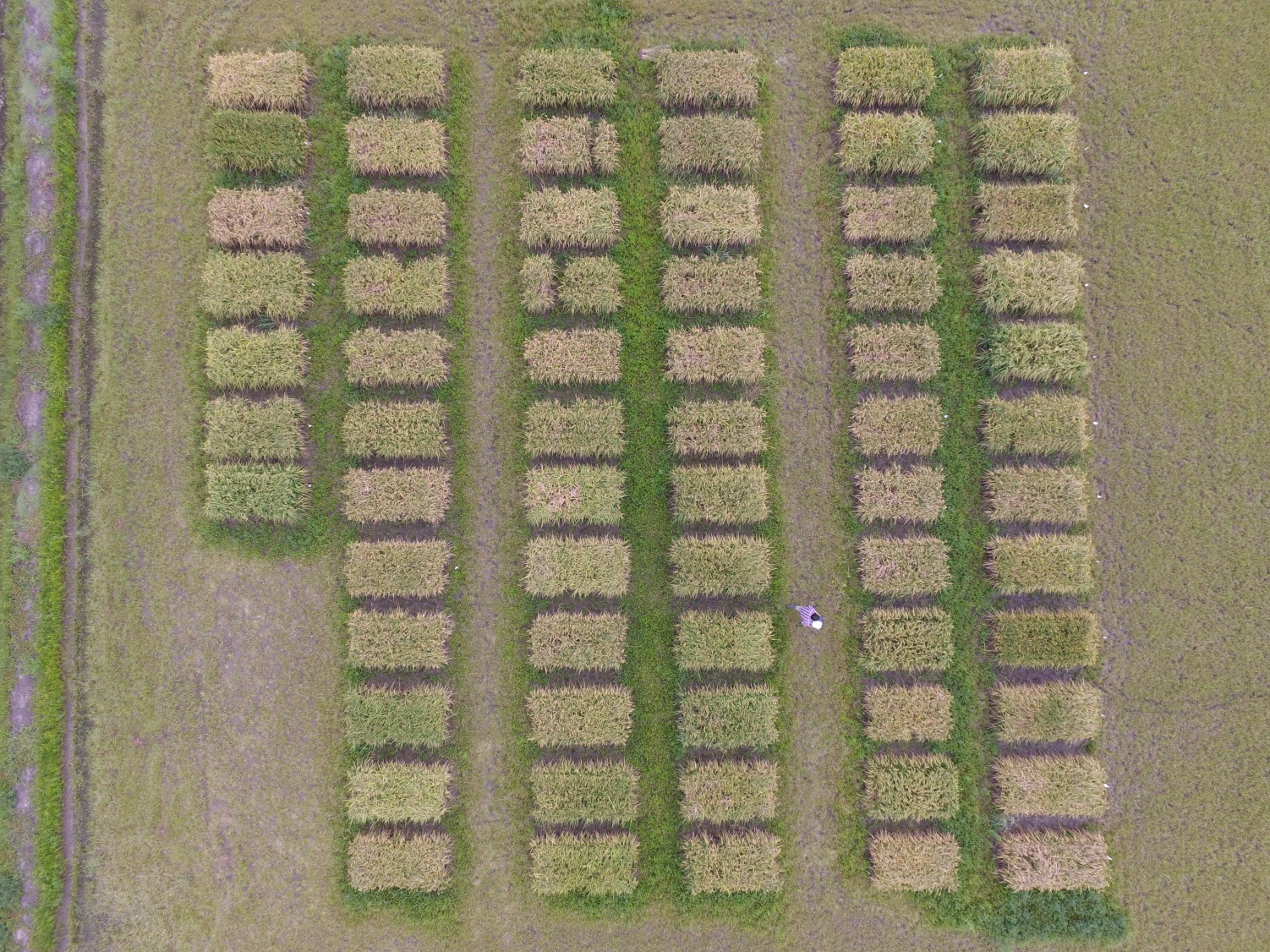

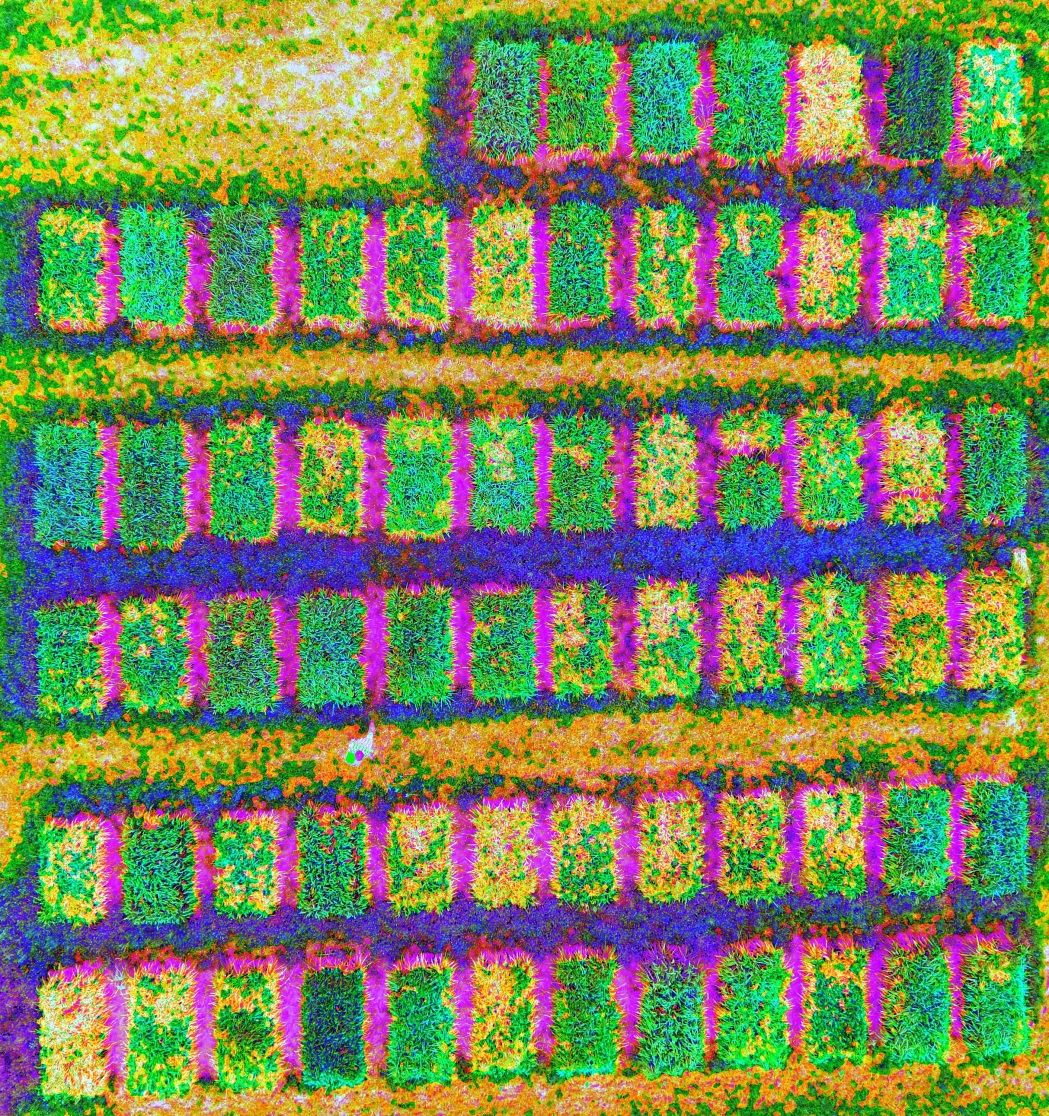


**（a） RGB image Second data collection （b） HLS image**

**
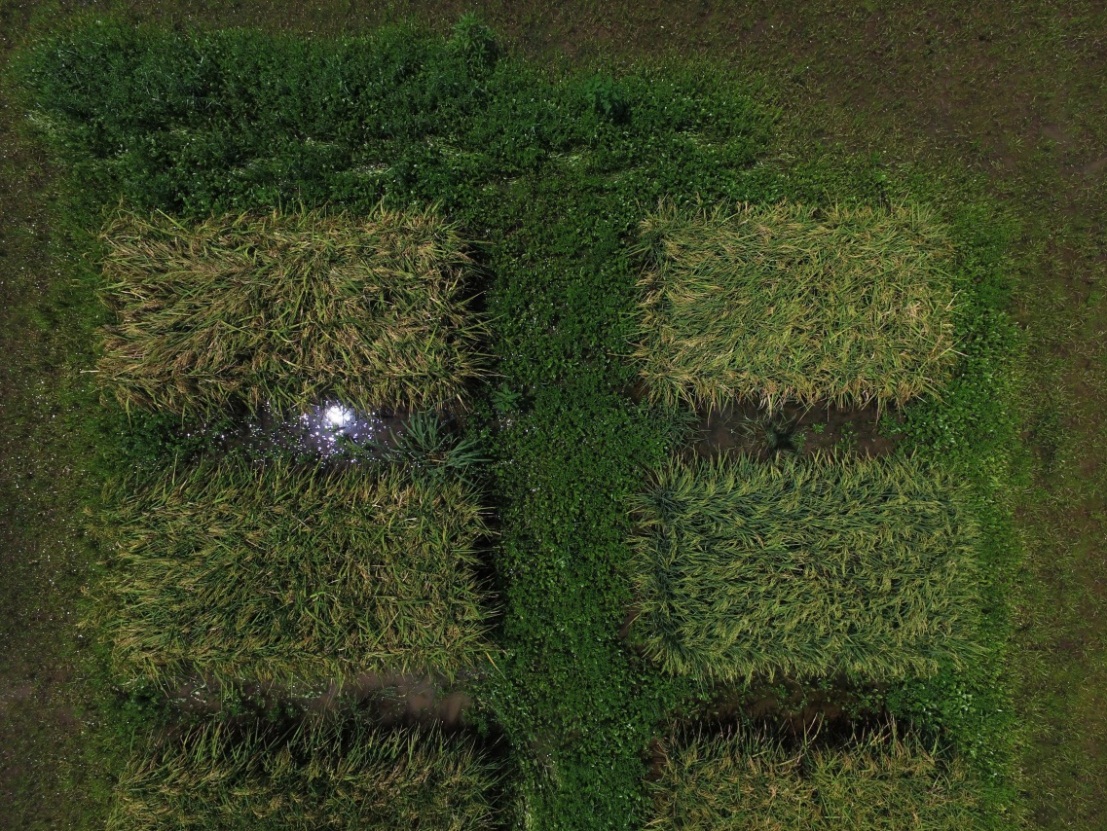

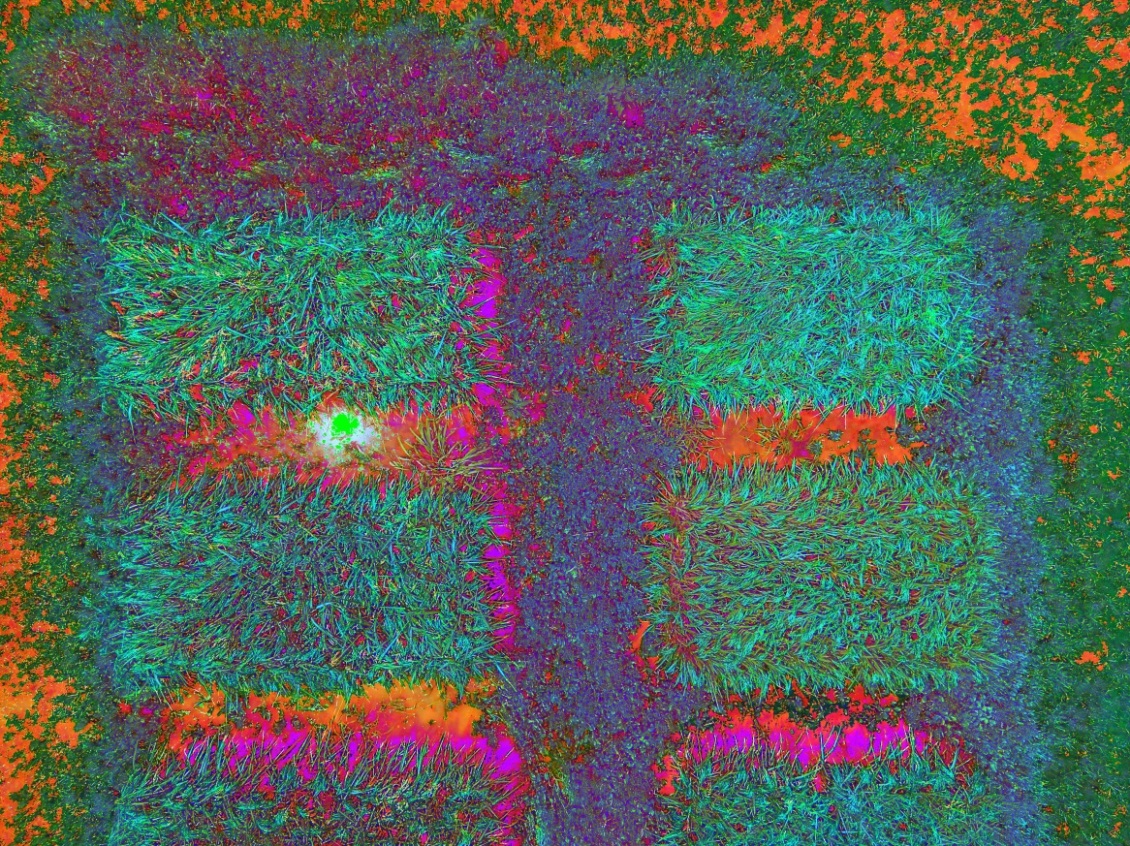
**

**（a） RGB image First data collection （b） HLS image**


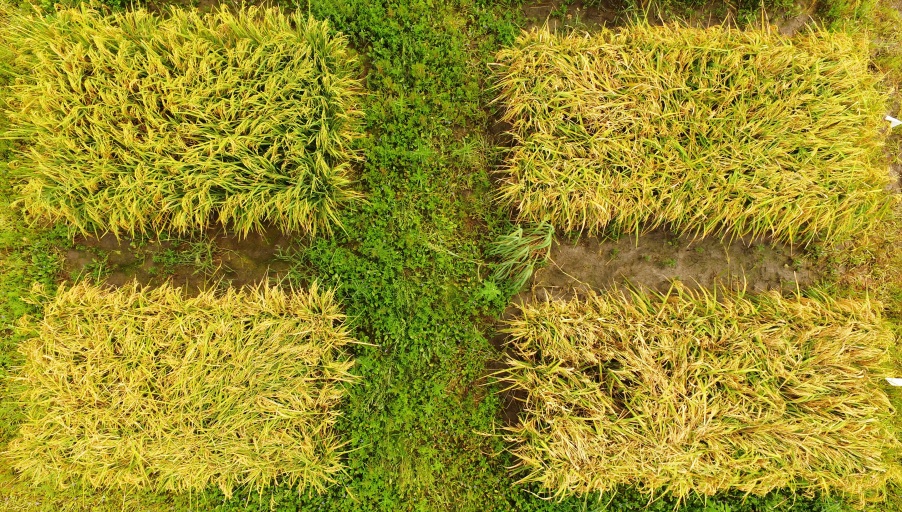

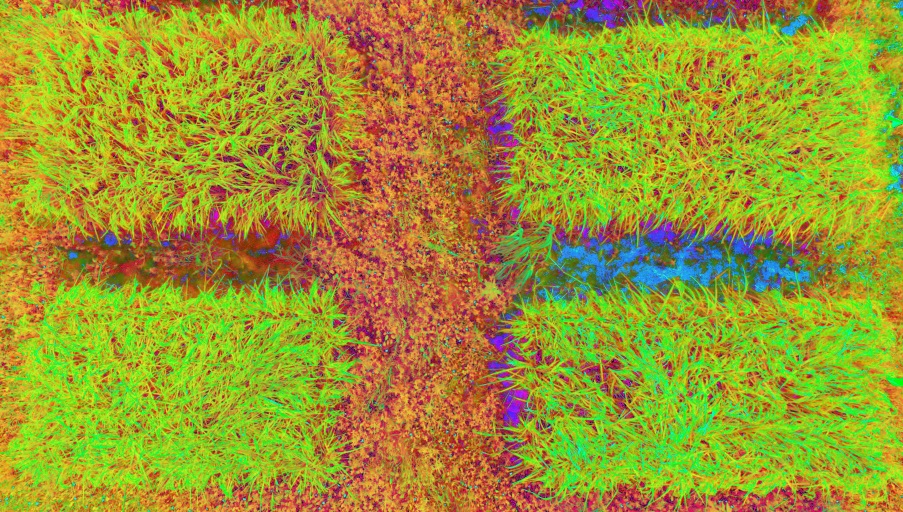


**（a） RGB image Second data collection （b） HLS image （BGR）**


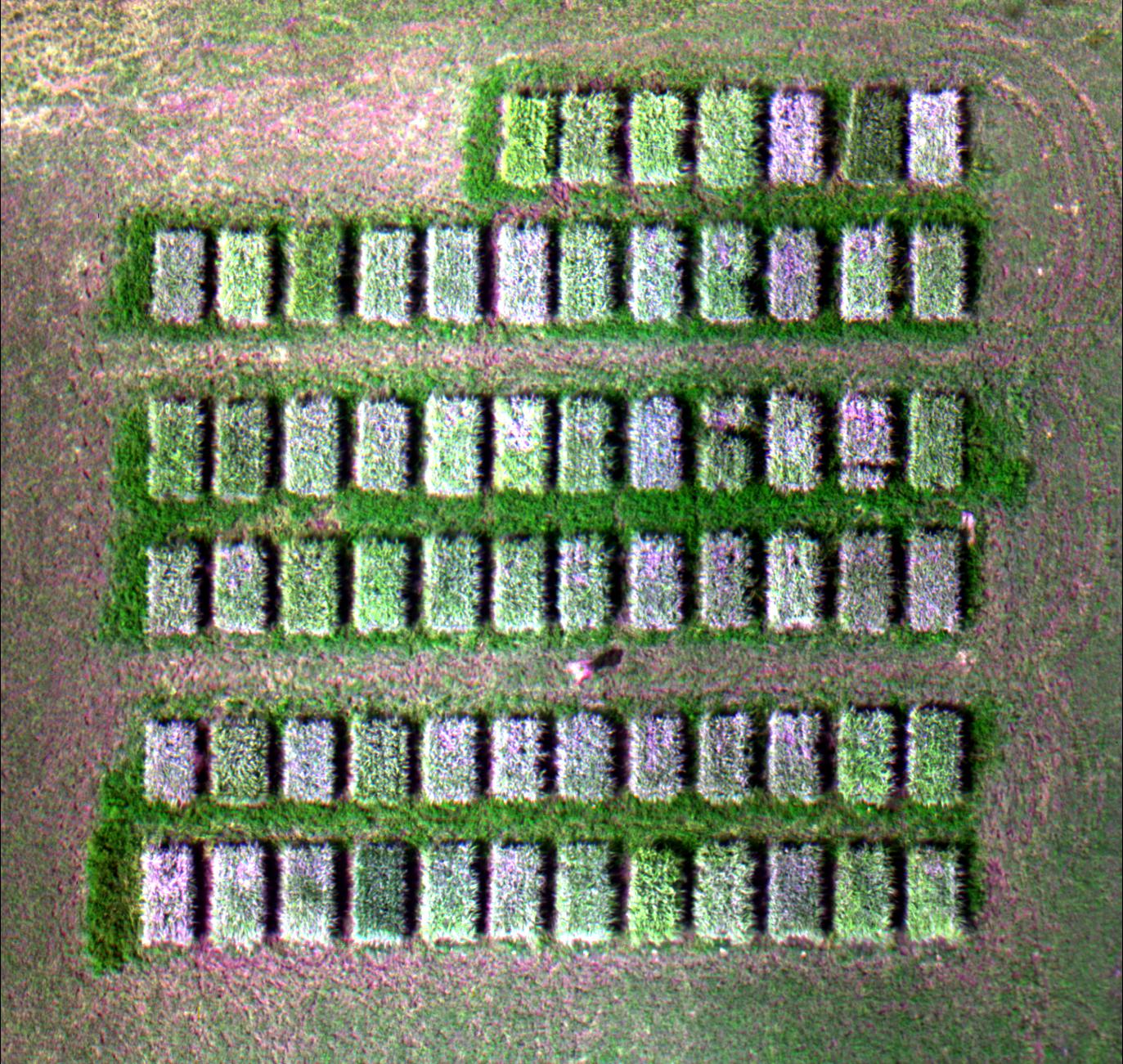

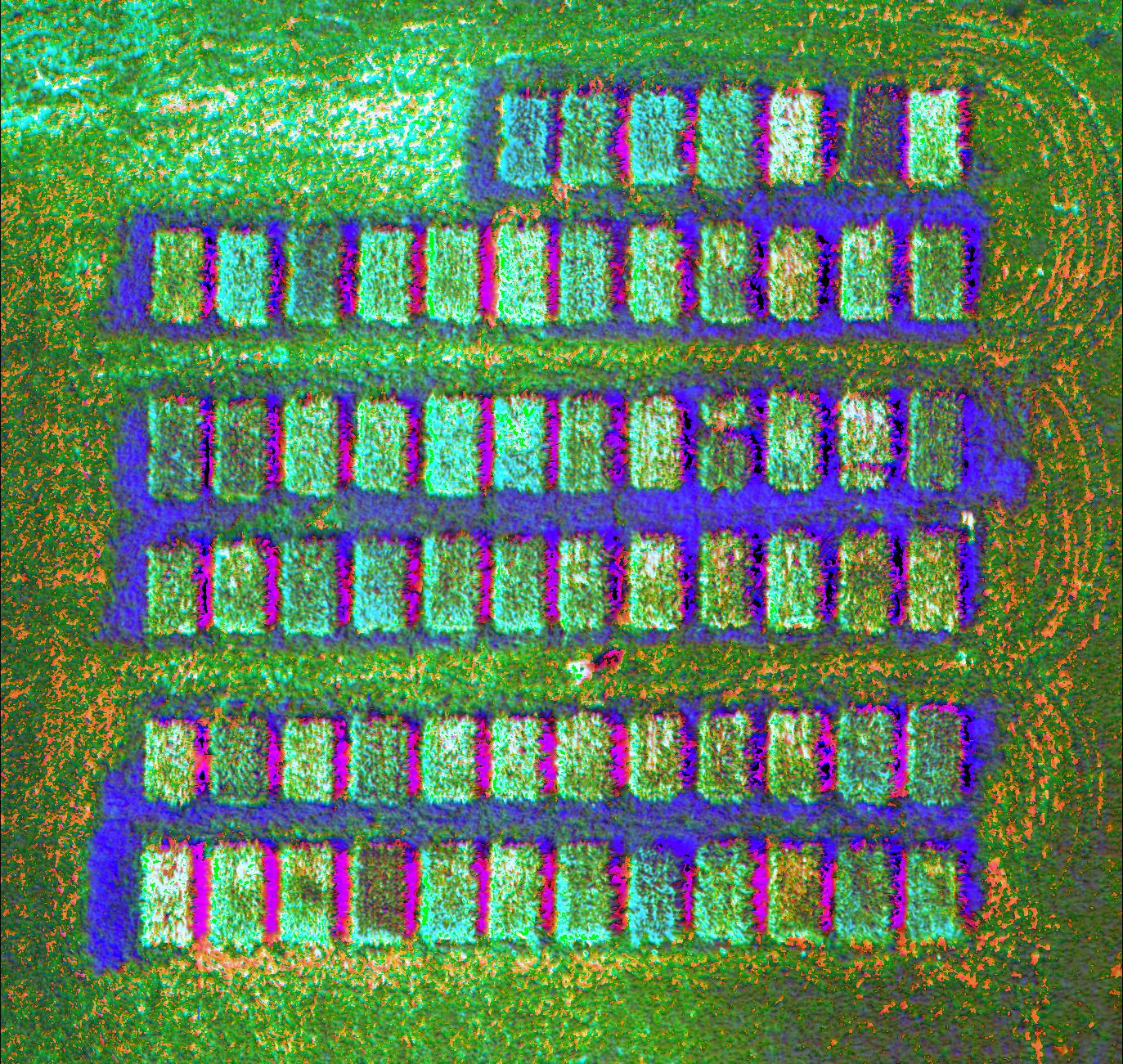


**（a） RGB image Second data collection （b） HLS image**


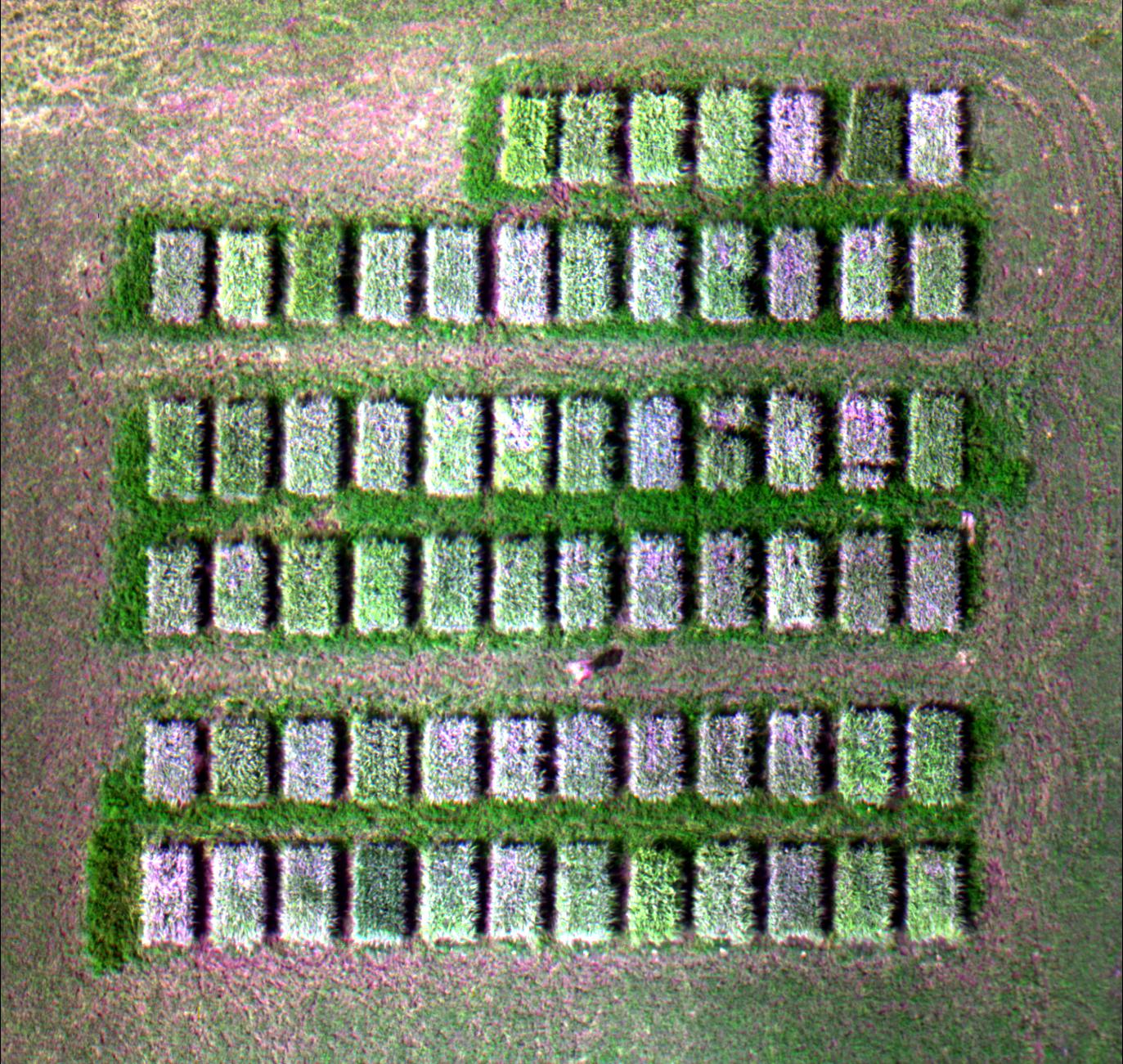

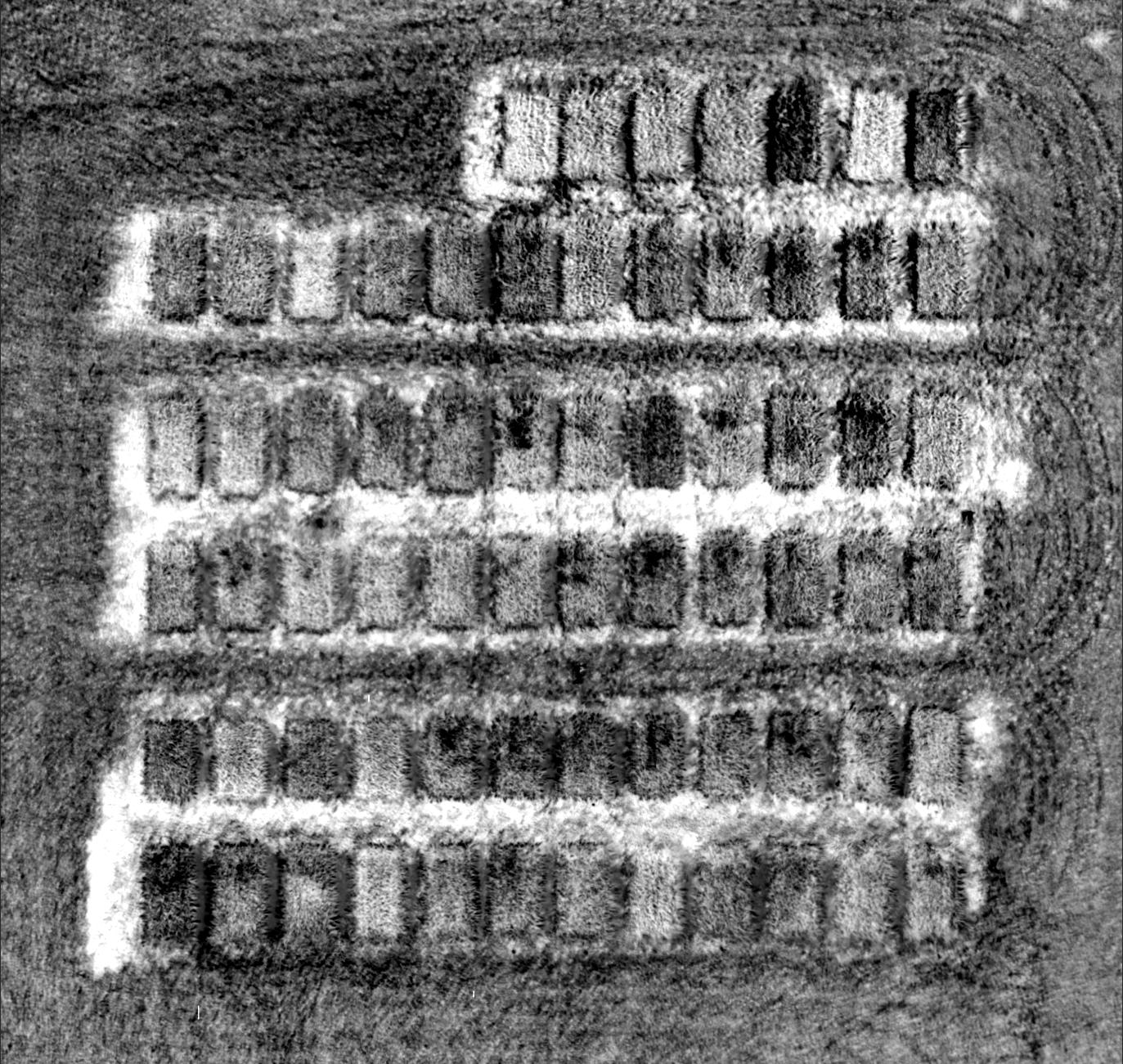


**Multiple spectra （Real color） Second data collection NDVI （Vegetation index）**
